# Cordycepin attenuates Salivary Hypofunction through the Prevention of Oxidative Stress in Human Submandibular Gland Cells

**DOI:** 10.7150/ijms.46707

**Published:** 2020-07-06

**Authors:** Atchara Jaiboonma, Palakorn Kaokaen, Nipha Chaicharoenaudomrung, Phongsakorn Kunhorm, Kajohnkiart Janebodin, Parinya Noisa, Paiboon Jitprasertwong

**Affiliations:** 1 School of Geriatric Oral Health, Institute of Dentistry, Suranaree University of Technology, Nakhon Ratchasima, Thailand.; 2 Laboratory of Cell-Based Assays and Innovations, School of Biotechnology, Institute of Agricultural Technology, Suranaree University of Technology, Nakhon Ratchasima, Thailand.; 3 Department of Anatomy, Faculty of Dentistry, Mahidol University, Bangkok, Thailand.

**Keywords:** cordycepin, salivary gland, xerostomia, saliva, dry mouth

## Abstract

Xerostomia (dry mouth) is a significant age-related condition. Meanwhile, cordycepin, the natural therapeutic agent, has demonstrated an anti-aging effect. Therefore, the present study aimed to investigate the preventive effects of cordycepin on secretory function in an *in vitro* model of hydrogen peroxide (H_2_O_2_)-induced salivary hypofunction. After being exposed to H_2_O_2_, human submandibular gland (HSG) cells were treated with various concentrations of cordycepin (6.25-50 µM) for 24, 48, and 72h. To evaluate cell proliferation and reactive oxygen species (ROS) generation, 3-(4, 5-dimethylthiazol-2-yl)-2, 5-diphenyltetrazolium bromide and 2, 7'-dichlorodihydrofluorescein diacetate assays were performed. The amylase activity was kinetically measured by 2-chloro-*p*-nitrophenol linked with maltotrioside. The expression of salivary, antioxidant and apoptotic markers at mRNA and protein levels were performed by reverse transcriptase polymerase chain reaction (RT-PCR) and immunofluorescence analysis, respectively. We demonstrated that cordycepin (6.25-25 µM) contributed to significant increases in expression of the salivary marker genes, *alpha-amylase 1 (AMY1A)* and *aquaporin-5 (AQP5),* and in amylase secretion without changes in cell viability. Under oxidative stress, HSG cells showed remarkable dysfunction. Cordycepin rescued the protective effects partially by decreasing ROS generation and restoring the expression of the salivary proteins, AMY and AQP5 via anti-oxidant and anti-apoptotic activity. In addition, the amount of amylase that was secreted from HSG cells cultured in cordycepin was increased. In conclusion, cordycepin demonstrated a protective effect on H_2_O_2_*-*induced HSG cells by decreasing ROS generation and upregulating the salivary function markers, *AMY1A* and *AQP5*, at both the transcriptional and translational levels.

## Introduction

Xerostomia, also referred to as dry mouth, is dryness of the oral cavity caused by a low salivary flow rate, lack of saliva and change in the chemical composition of saliva 1, 2. Therefore, salivary gland dysfunction or salivary hypofunction causes dry mouth, which can lead to several clinical conditions, including mastication problems and a decline in the quality of life, especially for patients who have received radiation as part of cancer therapy and elderly persons 3, 4. Furthermore, salivary hypofunction is defined as an objective reduction in the salivary flow rate involving physiological atrophy of the salivary gland, which manifests itself as morphological alteration in the gland 5, 6.

Reactive oxygen species (ROS) are one of main causes of oxidative stress, contributing to cell aging, and are strongly associated with age-related diseases 7. ROS and other free radicals are usually reduced by antioxidants in the body, the so-called oxidation-reduction regulatory mechanism; hence, the accumulation of ROS within the body is repressed 8, 9. However, antioxidant activity declines with age by interfering with the redox balance, resulting in protein denaturation, DNA damage, lipid peroxidation and apoptosis 10. Thus, oxidative stress can probably affect the secretory function of salivary glands in the elderly 11. Nevertheless, the relationship between age-related salivary hypofunction and oxidative stress has not been defined 12.

Cordycepin, also known as 3-deoxyadenosine, is one of the major bioactive compounds of *Cordycepes* sp., exerting various pharmaceutical properties (*e.g.* antitumor, anticancer and immunoregulatory effects 13, 14. Furthermore, the antioxidant activity of cordycepin has been recently studied 15. In addition, cordycepin could protect cells against oxidative stress, which induces cell damage. Cordycepin has also been demonstrated to inhibit mitochondrial injury and improve immune responses by scavenging ROS 16, 17. Previous studies have reported that cordycepin inhibits ROS generation and protects several cells (*i.e.* neuron and mesenchymal stem cells) from oxidative stress 18-20. Additionally, cordycepin could have antioxidant activity and attenuate oxidative stress *in vivo* 15.

However, the preventive effect of cordycepin on the dry mouth experimental model is still unclear. This research aimed to examine the effect of cordycepin on improvements in salivary secretion of H_2_O_2_-induced salivary hypofunction with xerostomia in human submandibular gland (HSG) cells *in vitro.*

## Materials and Methods

### Chemicals and reagents

All the chemicals and reagents used in this experiment, including cordycepin (MW=251.2 g/mol, 3-4,5-dimethyl-2-thiazolyl-2,5-diphenyl-2H-tetrazolium bromide (MTT), dimethyl sulfoxide (DMSO), 2',7'-dichlorodihydrofluorescein diacetate (DCFHDA), Hydrogen peroxide (H_2_O_2_) and amylase activity assay kits, were purchased from Sigma-Aldrich Co. (St. Louis, MO). Dulbecco's Modified Eagle Medium (DMEM), non-essential amino acids (NAAs) and foetal bovine serum (FBS) were purchased from HyClone (Logan, UT), whereas 0.5% trypsin-EDTA and L-glutamine were obtained from Gibco (Gibco, CA, USA).

### Cell culture

HSG cell line used in this study was obtained from the American Type Culture Collection (ATCC® HTB-41^TM^, Manassas, VA, USA). Cells were cultured in a DMEM medium supplemented with 10% (v/v) FBS, 1% (v/v) NAAs, 1% (v/v) L-glutamine and 1% (v/v) antibiotics (penicillin-streptomycin). Cells were cultured at 37 °C under an atmosphere of 5**%** CO_2_ with 95% humidity. The culture medium was replaced thrice a week.

### Determination of cell viability

Cell viability was evaluated by MTT assay. Briefly, HSG cells were seeded into 96-well tissue culture plates at a density of 1×10^4^ cells/well overnight, then the cells were treated in three different experiments; 1) treated with various concentrations of cordycepin (6**.**25-50 µM) for 24, 48, and 72 h; 2) treated with different concentrations of H_2_O_2_ (250-2000 µM) to induced cell toxicity for 30 min; 3) treated with H_2_O_2_ for 30 min and cordycepin ranging from 6**.**25-50 µM for 24 h. After each treatment, the cells were washed with 100 µl of 1× PBS, then 0.5 mg/ml of MTT solution in 1× PBS was added to each well and incubated at 37 °C for 4 h. After removal of the MTT solution, the formazan crystals were dissolved with 100 µl of DMSO, and the purple formazan solution was further measured for absorbance at 570 nm using a microplate reader (BMG Labtech, Ortenbreg). The percentage of cell viability was calculated according to the following equation:


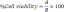


where A was the absorbance of OD of the treated group, and B was the absorbance of OD of the untreated group.

### Determination of ROS generation

Intracellular ROS generation was determined by DCFH-DA assay**.** Briefly, after the seeding of HSG cells, the cells were incubated with 500 µM H_2_O_2_ for 30 min to induce oxidative stress in cells, then washed with 1× PBS and added to cordycepin at various concentrations for 24 h. After being exposed to cordycepin, the cells were stained with 20 µM DCFH-DA at 37 °C for 30 min in the dark. Afterwards, the fluorescence intensity was measured at an excitation/emission of 485/535 nm using a fluorescence microplate reader (Thermo Scientific Varioskan, USA).

### RT-PCR analysis

After 24 h, total RNA was extracted from untreated and treated HSG cells in each well using the NucleoSpin RNA kit (Macherey-Nagel, Dueren, Germany), following the manufacturer's instructions. Complementary DNA was synthesized from 1 µg of total RNA using the ReverTra Ace qPCR Master Mix kit (Toyobo CO., LTD., Japan), according to the manufacturer's instructions. PCR analysis was performed to determine the expression of salivary-specific genes, *alpha-amylase 1 (AMY1A)* and *aquaporin-5 (AQP5)*, the expression of antioxidant genes, superoxide dismutase *(SOD1)*, *catalase (CAT)*, *glutathione peroxidase (GPX1)*, and the expression of apoptotic genes, *caspase-3 (CASP-3)* and *B-cell leukemia/lymphoma 2 (BCL-2).* The mRNA levels of *glyceraldehyde-3-phosphate dehydrogenase (GAPDH)* were employed as internal controls. The primer sequences and RT-PCR conditions were shown in Table 1. The PCR products were identified by electrophoresis on 1.5% agarose gel and visualized by ethidium bromide staining. The mRNA band density of each gene was analyzed and quantified using densitometer and ImageJ software from the NIH website and shown as the mean ± SD of the results from three independent experiments. Each band image was calculated for the total band density. The relative density of genes of interests and *GAPDH* was calculated by dividing the density of each gene by the density of *GAPDH* of the same sample. Lastly, the relative gene expression for the treated group was plotted as a fold-change normalized to the untreated control.

### Immunofluorescence staining

Cells were grown on the surface of coverslips in 24-well plates until a confluence of 80%. After being treated with cordycepin, cells were fixed with 4% paraformaldehyde (PFA) solution for 30 min at room temperature and rinsed with 1× PBS twice. Cells were permeabilized and blocked in blocking buffer containing 1% bovine serum albumin (BSA), 1% Triton X-100 of 1× PBS at 4 °C for 1 h for amylase and 4% BSA, 0.1% Triton X-100 of 1× PBS at 4 °C for 30 min for AQP5. After cell permeabilization, the cells were stained into each well, then incubated overnight at 4 °C with anti-alpha-amylase (anti-AMY) antibody (1:100 dilution; Santa Cruz Biotechnology Inc.) and ant-aquaporin-5 (anti-AQP5) antibody (1:100 dilution; Sigma-Adrich Co.), then washed with 1× PBS twice. After being washed with PBS, the cells were incubated at room temperature for 1 h with Rhodamine Red-X goat anti-mouse immunoglobulin G (IgG) 1:500 dilution; Invitrogen) for anti-AMY or AlexaFluor 488 goat anti-rabbit IgG (1:1000 dilution; Invitrogen) for anti-AQP5. Lastly, the cells were incubated with 4 µl of 4'6-diamidino-2-phenylindole (DAPI) (Invitrogen) for nuclear staining and observed under a ZOE™ Fluorescent Cell Imager (Bio-rad, CA, USA). The fluorescence intensity was quantified by ImageJ software. With RGB images, results are calculated using brightness values by the software will convert the RGB pixels to brightness values. To rule out the bias during the determination, we selected two areas of interest with approximately similar cell numbers represented by DAPI nuclear staining. All measurements of each image were set with the same parameters including the subtraction of fluorescence background. The fluorescence intensity was presented as a fold change which was relative to the untreated (control) group.

### Amylase secretion

Amylase secretion was measured by biochemical assay. After cell culture periods, the culture medium was collected from each well. Amylase secretion was measured using the amylase activity assay kit (Sigma-Andrich Co.), according to the manufacturer's protocol. Amylase activity was measured using a microplate reader at 405 nm. The kinetic reaction of amylase activity was calculated from the change in absorbance per 30 s for 2 h.

### Statistical analysis

Data were presented as the means ± SDs of three independent measurements (n=3). All analyses were performed using the Statistical Package for the Social Sciences (SPSS) software 23.0 for Windows. For comparison of the treatments, statistical significance was assessed by student's t-test to compare means from two independent sample groups at *P-value* (*p*<0.05).

## Results

### Cordycepin prevents against H_2_O_2_-induced toxicity in HSG cells

**Figure 1:**
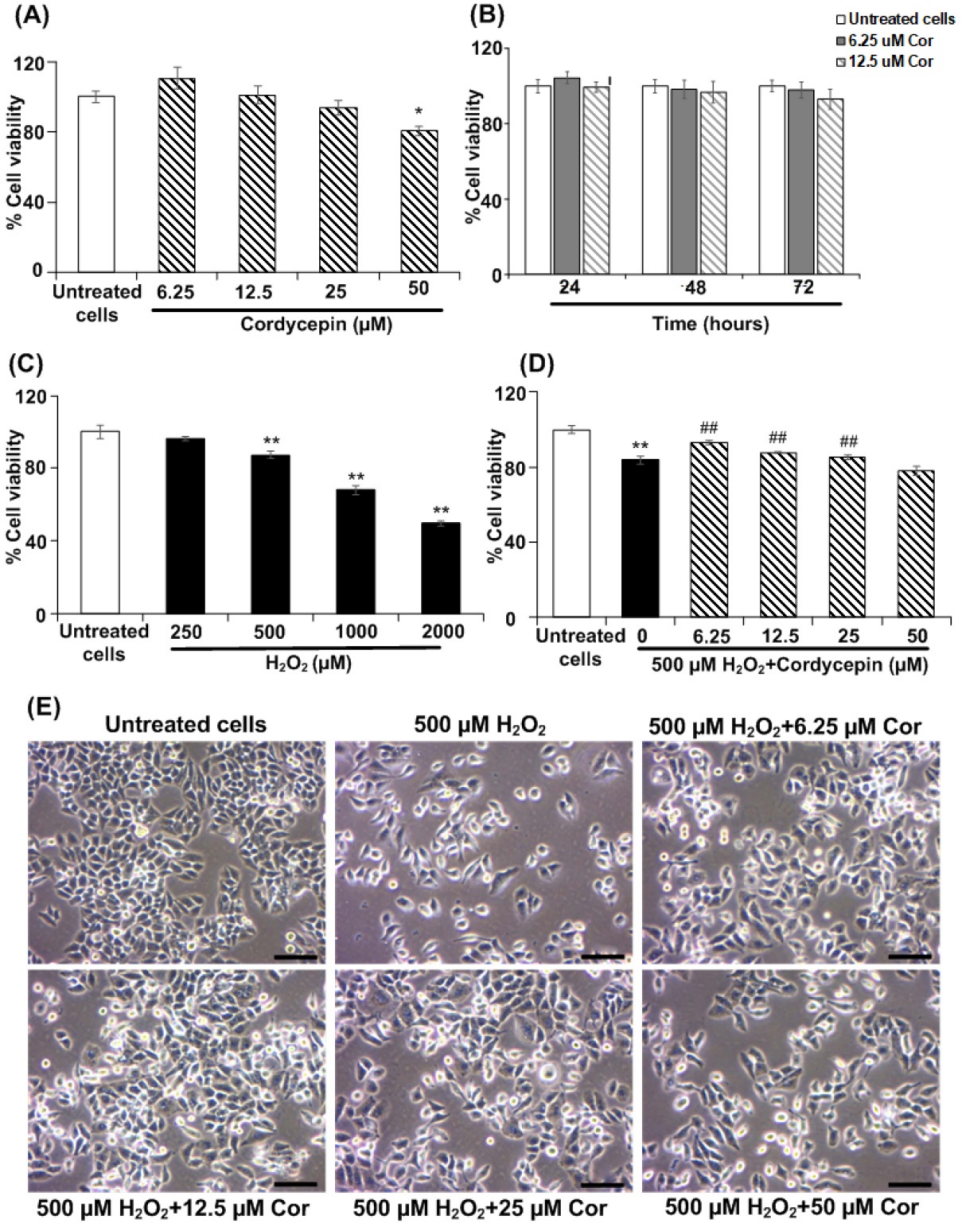
**Cordycepin prevented HSG cells from H_2_O_2_-induced cell toxicity.** Cells were treated with various concentrations of cordycepin (6.25-50 µM) for 24, 48 and 72 h (**A,B**) and different concentrations of H_2_O_2_ (250-2000 µM) were used to induced cell toxicity for a 30 min culture period (**C**). The post-treatment of HSG cells under 500 µM H_2_O_2_ for 30 min and cordycepin ranging from 6.25-50 µM for 24 h was measured by MTT assay (**D**). Cell morphology of HSG cells exposed to H_2_O_2_ and cordycepin under an inverted microscope (**E**). The scale bar represents 20 µm. *p<0.05 and **p<0.01 compared to the untreated group, and ^##^*p*<0.01 compared to the H_2_O_2_ group.

In this study, we first performed the cytotoxicity test of cordycepin on HSG cells. The HSG cells were treated with various concentrations of cordycepin (6**.**25-50 µM) for 24, 48, and 72 h and then measured by MTT assay. As shown in Figure 1A, the HSG cells exposed to most concentrations of cordycepin at 24 h did not show their viability alteration. Nevertheless, the highest cordycepin concentration (50 μM) was slightly toxic in HSG cells (19.66% ± 2.45). These suggests that cordycepin especially at low concentrations did not display a toxic effect to the HSG. In addition, the cell viability at different time points (24, 48, and 72 h) showed that % cell viability of HSG remains unchanged at various time points, indicating that cordycepin had no proliferative effects on HSG cells (Figure 1B). The percentage of viable HSG cells was considerably decreased upon exposure to H_2_O_2_ (Figure 1C), indicating cytotoxicity. Furthermore, we investigated the preventive effects of cordycepin against H_2_O_2_-induced cell toxicity. The post-treatment group with cordycepin under H_2_O_2_ revealed significantly increased cell viability when compared to the H_2_O_2_ group (Figure 1D). Moreover, the post-treatment group with cordycepin in HSG cells showed cell morphology similar to that of the untreated group (Figure 1E).

### Cordycepin promotes the expression of salivary genes in H_2_O_2_-induced HSG cells

**Figure 2:**
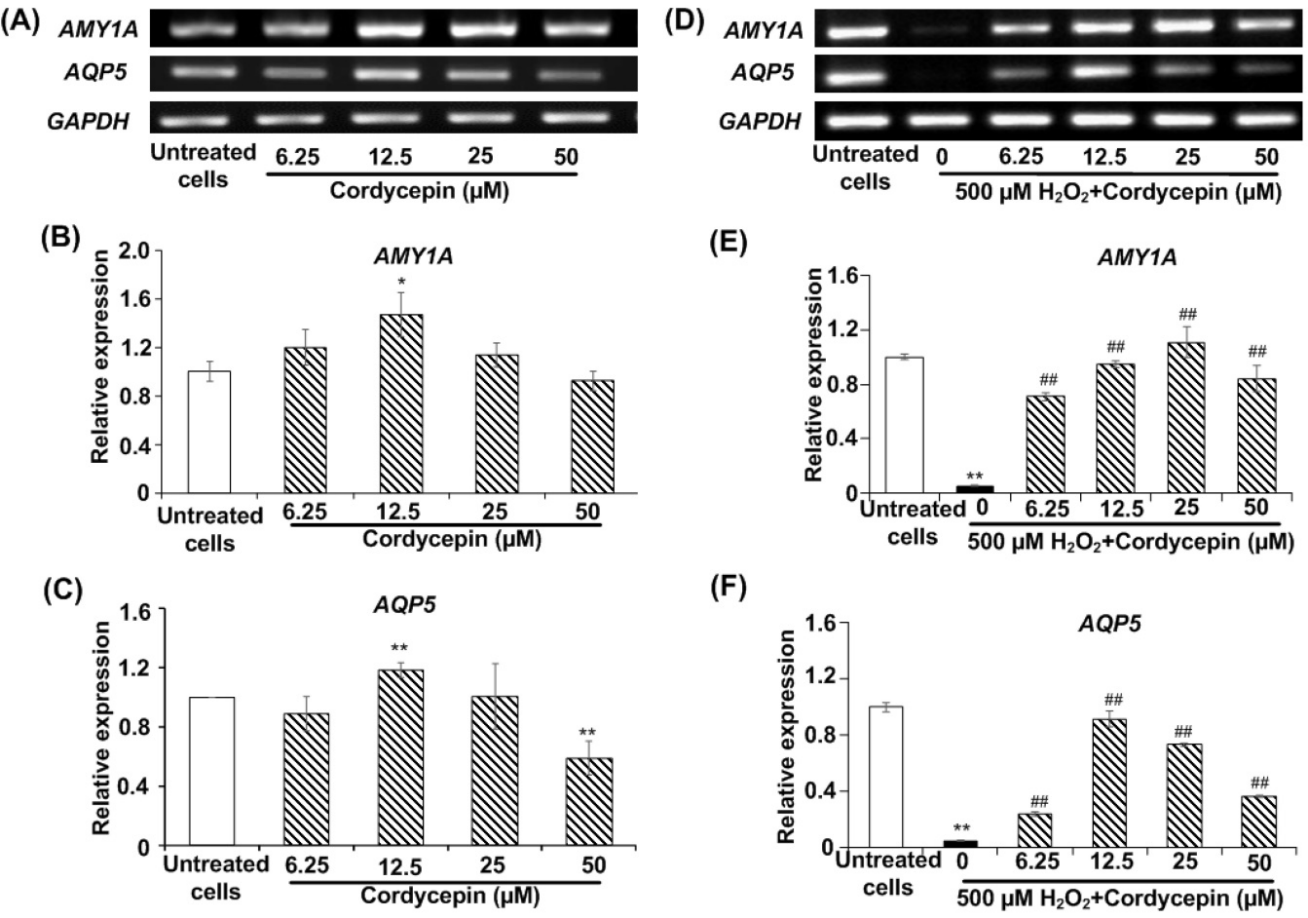
** Cordycepin upregulated salivary marker genes in H_2_O_2_-induced HSG cells.** Cells were treated with cordycepin ranging from 6.25 µM to 50 µM for 24 h. The mRNA expression for *AMY1A* and *AQP5* were analysed by RT-PCR (**A-C**). Cordycepin promoted *AMY1A* and *AQP5* expression in HSG cells exposed to H_2_O_2_ for 30 min (**D-F**). The relative mRNA expression levels of *AMY1A* (**B-E**) and *AQP5* (**C-F**) genes were evaluated by image J NIH software and normalized with *GAPDH* gene. Gel electrophoresis results are from one representative experiment and bar charts are derived from analysis of relative expression from three independent experiments. *p<0.05 and **p<0.01 compared to the untreated group, and ^##^*p*<0.01 compared to the H_2_O_2_ group.

HSG cells were cultured in each concentration of cordycepin treatment for a 24 h period; salivary gene expression was then comparatively assessed. The salivary-specific gene expression, *AMY1A* and *AQP5*, of the cultured HSG cells was determined by RT-PCR analysis. The band intensities of mRNA expression of *AMY1A* and *AQP5* in each cordycepin concentration-treated HSG cells were demonstrated (Figure 2A). In cordycepin concentrations (6.25, 12.5, 25 µM), the relative expression of *AMY1A* gradually increased as compared to that found in the untreated group. In particular, 12.5 µM of cordycepcin significantly increased *AMY1A* expression (Figure 2B). The expression of *AQP5* detected in the 12.5 µM of cordycepin group was also higher than that detected in the untreated group (Figure 2C). Interestingly, the increase in salivary-specific gene expression observed among the cells cultured in the cordycepin treatments were much different from one another. In addition, cordycepin had protective effect on H_2_O_2_-induced HSG cell dysfunction, the gene expression demonstrated that all cordycepin concentrations significantly increased the levels of *AMY1A* and *AQP5* in H_2_O_2_-induced HSG cells compared to the induced cells without the cordycepin treatment (Figures 2D-F), suggesting that cordycepin could rescue the salivary function after oxidative stress exposure).

### Cordycepin alters both AMY and AQP5 protein expressions in H_2_O_2_-induced HSG cells

**Figure 3:**
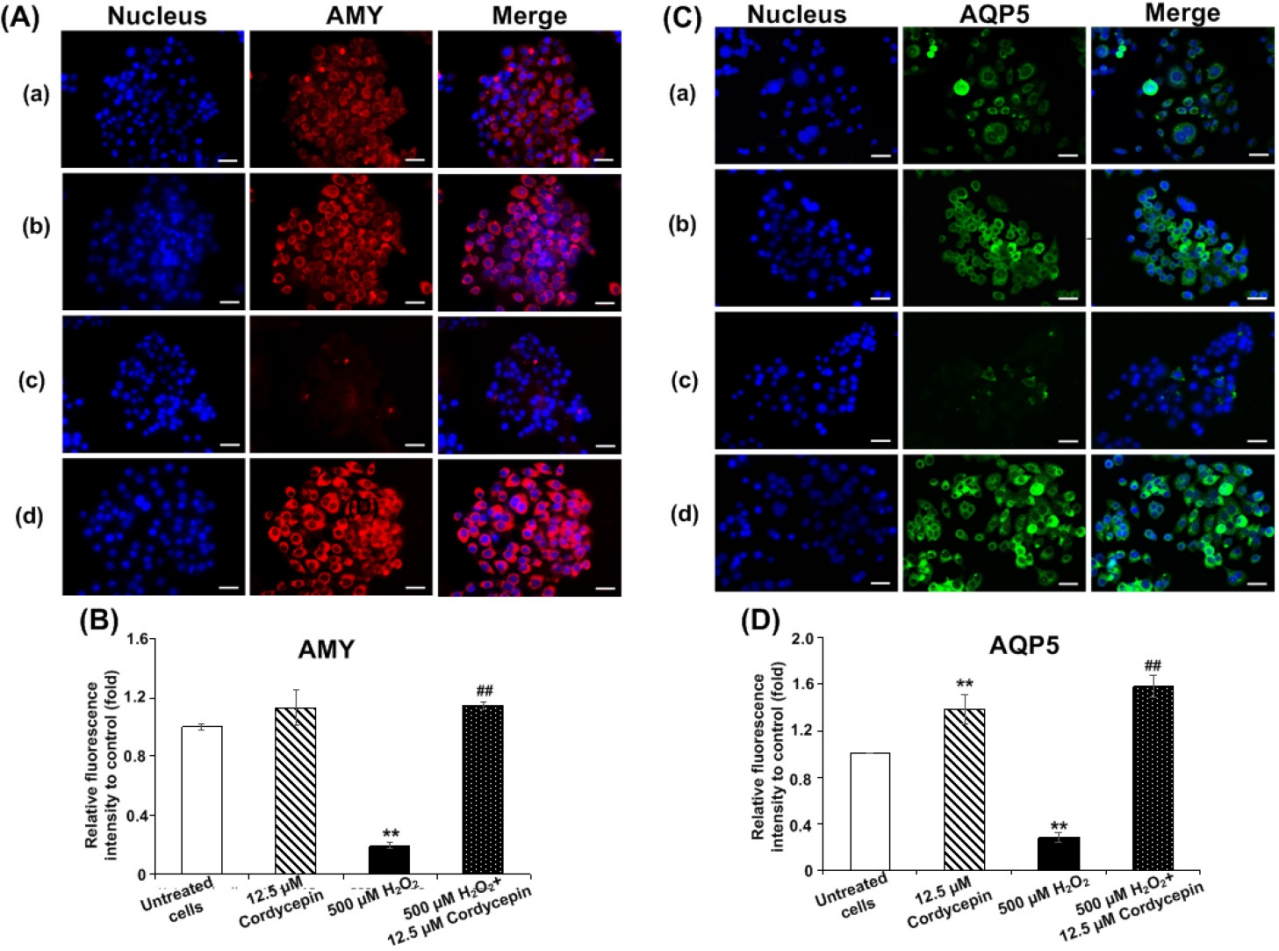
**Cordycepin rescued the expression of salivary proteins in H_2_O_2_-induced HSG cells.** Both AMY (**A**) and AQP5 (**C**) protein expression in HSG cells were determined by immunofluorescence. Cells were treated with cordycepin at either 0 µM (a) or 12.5 µM (b) for 24 h, treatment with 500 µM H_2_O_2_ for 30 min without (c) or with 12.5 µM cordycepin for 24 h (d). The relative fluorescence intensity of both AMY (**B**) and AQP5 (**D**) proteins in cordycepin-treated HSG cells were normalized with the untreated group. Immunofluorescence images are from one representative experiment. Bar charts were expressed as mean ± SD from three independent experiments. The scale bar represents 40 µm. **p<0.01 compared to the untreated group, and ^##^*p*<0.01 compared to the H_2_O_2_ group.

The expression of AMY and AQP5 proteins in HSG cells under H_2_O_2_-induced oxidative stress was investigated using immunofluorescence assay. The effect of cordycepin stimulation on AMY protein expression was indicated (Figure 3A). The relative fluorescence intensity revealed that no significant differences were observed in HSG cells treated with either cultured medium or cordycepin. Intriguingly, H_2_O_2_-induced oxidative stress revealed a significant reduction in AMY protein expression when compared to the untreated group. Compared to the H_2_O_2_ group, the post-treatment group with cordycepin showed a dramatic increase of AMY protein expression in HSG cells (Figure 3B). The expression of AQP5 protein in HSG cells treated with either cordycepin, H_2_O_2_ or cordycepin post-treatment was demonstrated (Figure 3C). The relative fluorescence of AQP5 was also significantly increased in HSG cells treated with cordycepin. However, compared to the untreated group, the AQP5 protein expression of H_2_O_2_-induced HSG cells was extremely decreased. Noticeably, the cells post-treated with cordycepin were found to have significantly increased levels of AQP5 protein, indicating that under H_2_O_2_-induced oxidative stress, cordycepin could stimulate the expression of both AMY and AQP5 (Figures 3B & D). These results suggest that cordycepin could rescue both AMY and AQP5 protein expressions under H_2_O_2_-induced oxidative stress in HSG cells.

### Cordycepin attenuates against intracellular ROS generation and affects apoptotic gene expression in H_2_O_2_-induced HSG cells

**Figure 4:**
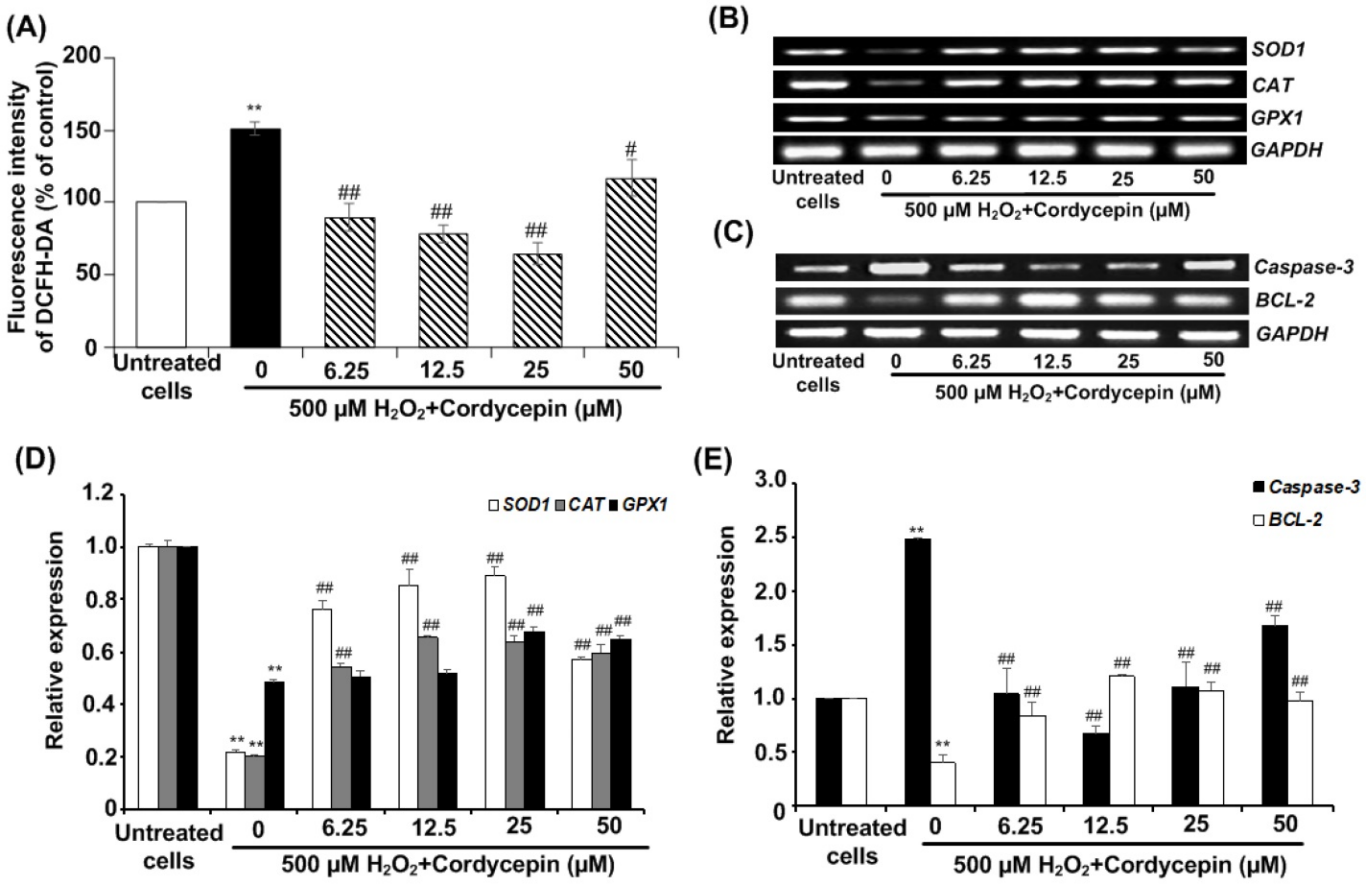
**Cordycepin attenuated H_2_O_2_-induced intracellular ROS generation in HSG cells.** Cells were induced with 500 µM H_2_O_2_ for 30 min and exposed to cordycepin ranging from 6.25-50 µM for 24 h. The relative fluorescence intensity of DCFH-DA was determined by DCFH-DA assay (**A**). The mRNA expression for antioxidant genes, *SOD1*, *CAT*, and *GPX1* (**B**) and apoptotic genes including *caspase-3* and *BCL-2* (**C**) were analysed by RT-PCR. The relative mRNA expression levels of *SOD1*, *CAT* and* GPX1* (**D**), *Caspase-3* and *BL-2* (**E**) genes were evaluated by image J NIH software and normalized with *GAPDH.* Gel electrophoresis are from one representative experiment and bar charts are derived from analysis of relative expression from three independent experiments. **p<0.01 compared to the untreated group; ^#^*p*<0.01 and ^##^*p*<0.01 compared to the H_2_O_2_ group.

Next, we investigated the effect of cordycepin against H_2_O_2_-induced intracellular ROS generation in HSG cells using DCFH-DA fluorescence assay. The fluorescence intensity of DCFH-DA was considerably increased by 500 µM H_2_O_2_ when compared to the untreated group, indicating that H_2_O_2_ enables to generate intracellular ROS. However, the post-treatment with cordycepin effectively reduced intracellular ROS generation in HSG cells (Figure 4A). To confirm the preventive effects of cordycepin in HSG cells, the expressions of antioxidant genes *(i.e. SOD1*, *CAT* and *GPX1)* and apoptotic genes were evaluated. The band intensities of mRNA expression of these antioxidant genes were upregulated in HSG cells cultured in each concentration of cordycepin post-treatment (Figure 4B & D). The relative expression of *CAT* and *SOD1* were increased significantly in all concentrations of cordycepin whereas that of *GPX1* were increased significantly in certain concentrations as compared to that found in the untreated group (Figure 4D). Similarly, we also found that, H_2_O_2_ induced up-regulation of apoptotic gene, *caspase-3* and down-regulated *BCL-2* gene expression. Significantly, a decrease in the level of *caspase-3* and an increase in *BCL-2* in H_2_O_2_-induced HSG cells after post-incubation with cordycepin were demonstrated (Figure 4C & E). This may indicate the anti-apoptotic activity of cordycepin on H_2_O_2_-induced HSG cells.

### Cordycepin stimulates the amylase secretion from HSG cells under H_2_O_2_-induced oxidative stress

**Figure 5:**
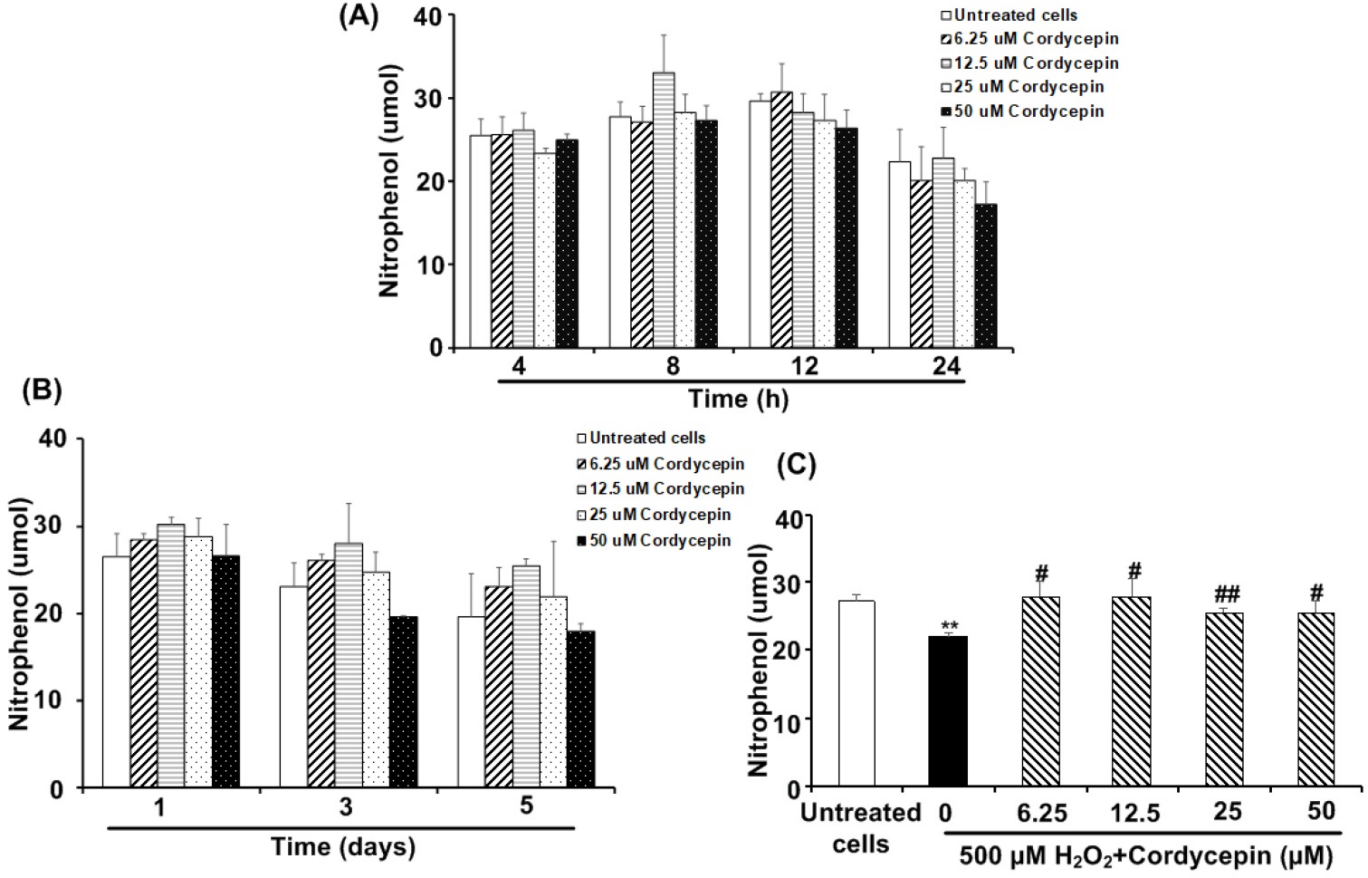
**Cordycepin increased amylase secretion in H_2_O_2_-induced HSG cells.** Cells were treated with various concentrations of cordycepin for short-term culture at 4, 8, 12 and 24 h (**A**) and long-term culture at 1, 3 and 5 days (**B**). Cordycepin increased amylase secretion in HSG cells exposed to H_2_O_2_ for 30 min (**C**). Amylase activity was kinetically measured in a cultured medium of HSG cells exposed to H_2_O_2_ without or with cordycepin treatment for 24 h. Data are represented as mean ± SD (n=3). **p<0.01 compared to the untreated group, and ^#^*p*<0.05 and ^##^*p*<0.01 compared to the H_2_O_2_ group.

The amounts of amylase secreted from the HSG cells cultured in each cordycepin concentration were reported (Figure 5). Compared to the untreated group, specific concentrations of cordycepin treatment could stimulate the cells to secrete more amylase after short and long-term culture periods. For the short-term culture periods, cordycepin stimulated amylase secretion, with the high levels during 812 h. The 12.5 µM of cordycepin treated HSG cells showed the highest level of amylase secretion at 8 h. However, at 24 h, the secretion of amylase was dropped in all groups (Figure 5A). It is also noted that amylase secretion from the cells cultured in 6.25, 12.5 and 25 µM of cordycepin (1-5 days) were increased but statistically insignificant (Figure 5B). To further study the effect of cordycepin in amylase secretion of HSG cells, cordycepin was used to treat H_2_O_2_ -exposed HSG cells. As expected, after 30 min of H_2_O_2_ treatment, the amylase secretion in the culture medium was suddenly decreased. Surprisingly, the amount of amylase was significantly increased in the culture medium when cordycepin was used for post-treatment (Figure 5C).

## Discussion

Xerostomia is a common side effect of numerous medications and other therapies, especially found in patients that have received radiotherapy for the treatment of head and neck cancer and in elderly individuals 21, 22. Current treatments for xerostomia include salivary substitutes, hyperbaric oxygen and gene therapy as well as salivary stimulants such as pilocarpine (a non-selective muscarinic receptor agonist) 23-25. However, these treatments still have the potential risk for undesirable adverse effects 26. The major bioactive compound of *Cordyceps* sp. is cordycepin, also known as 3-deoxyandenosine, which has various pharmaceutical properties including antitumor, anticancer, anti-inflammatory, immunomodulatory and antioxidant activities 13, 14, 27. This study indicated that cordycepin could stimulate the gene and protein expression of salivary markers and amylase secretion, while it reduced intracellular ROS generation in HSG cells via anti-oxidant and anti-apoptotic activities. The protective effect of cordycepin could enhance the H_2_O_2_-induced HSG cells to survive and revive the functions as a vital saliva-producing cells.

Amylase (AMY) and aquaporin-5 (AQP5) are two important specific markers for functional salivary glands 28. Amylase, which is encoded by *AMY1A* gene, is one of the major protein components of saliva and is a digestive enzyme for calcium binding responsible for starch hydrolysis 29-31. In addition, aquaporin-5, which is encoded by *AQP5* gene, is a water-selective channel protein expressed in the apical membrane of epithelial cells of serous acini in the salivary glands 32-35. The osmolality and the concentration of electrolytes in salivary fluid secretion are directly controlled by AQP5 protein. In this study, cordycepin enhanced the expression of both the *AMY1A* and *AQP5* genes in HSG cells. Although the effective dose to stimulate *AMY1A* (25 µM) and *AQP5* (12.5 µM) genes was different, it did not show a much difference in a fold-change expression. The concentration at 12.5 µM of cordycepin was chosen for further study since we expected the most effective result with the lower concentration of cordycepin. This concentration showed an increasing effect in *AMY1A* and *AQP5* for both gene and protein levels. This is promising since the previous study showed that AQP5 is one of key proteins to cause xerostoma. In addition, a study has previously reported that high expression of *AQP5* leads to an increase in a salivary amylase level in diabetes induced xerostomia rats 36.

Salivary gland hypofunction and reduced expression of *AQP5* are the oxidative stresses leading to xerostomia 2. Our results showed that cordycepin could promote the expression of *AQP5* genes, suggesting that the water channels are opened by cordycepin treatment after administration of H_2_O_2_. During the study of the expression of protein levels, the reduction in expression of AMY and AQP5 protein levels have been observed in HSG cells exposed to H_2_O_2_-induced oxidative stress. However, AMY and AQP5 protein levels were significantly increased in cordycepin-treated HSG cells after H_2_O_2_ administration. These results suggested that the oxidative stress related with xerostomia-induced hyposalivary function can be reversed effectively by treatment with cordycepin. Previous studies have proposed that the mechanism of cordycepin in salivary gland cells might involve several pathways, such as sodium/hydrogen exchanger-1 (NHE1) activity 37, c-Jun N-terminal kinase (JNK) phosphorylation 38 and protein disulphide isomerase (PDI) 39.

ROS generation is a major cause of oxidative stress, which is harmful to the biomolecules inducing the pathology in the body, causing age-related cell damage. Oxidative stress is a main cause of cellular damage in several diseases, including xerostomia 40, 41. In addition to therapeutic interventions, bioactive compounds with antioxidant activity have been reported to be capable of protecting cells from oxidative stress 42, 43. The bioactive compound cordycepin has been previously reported to be effective against oxidative and endoplasmic reticulum stress by reducing intracellular ROS generation* in vitro* 15, 44. Wang et al. revealed that cordycepin could prevent human bone marrow mesenchymal stem cells (BM-MSCs) from H_2_O_2_-induced inhibition of osteogenesis 18. Moreover, cordycepin protected against neuron cell death in PC12 and C6 glial cells from oxidative stress-induced neurotoxicity 19, 20. In animal models, cordycepin treatment decreased oxidative damage and boosted the differentiation of BM-MSCs 18. To examine cells under oxidative stress, intracellular ROS generation and the expression of antioxidant genes, which are widely used as markers for oxidative stress, were determined. In this study, we observed that ROS generation was increased by H_2_O_2_-induced oxidative stress in HSG cells; nevertheless, it was effectively alleviated by cordycepin treatment. To confirm intracellular ROS generation, we determined the effect of cordycepin through the antioxidant gene expression, *SOD1*, *CAT* and *GPX1* in the H_2_O_2_-induced HSG cells. SOD1 is an important endogenous antioxidant enzyme. It catalyzes the dismutation of superoxide anion free radicals to hydrogen peroxide and oxygen and acts as the first enzyme in defense of cell death from ROS 45. CAT is used in the reduction of hydrogen peroxide to water and oxygen, while GPX1 is the intracellular enzyme used to reduce hydrogen peroxide to water and lipid peroxides 46. Our results showed that the post-treatment with cordycepin considerably upregulated the expression of the *SOD1*, *CAT* and *GPX1* genes in HSG cells induced by H_2_O_2_. Furthermore, we also demonstrated that, post-treatment with cordycepin significantly decreased the expression of *caspase-3*, but increased *BCL-2* expression in H_2_O_2_-induced HSG cells. Taken together, these results indicated that cordycepin could effectively block ROS generation through the anti-oxidant and anti-apoptotic activities.

The composition of saliva is 99% water and 1% solids and protein, and it produced by the salivary glands consisting of the parotid, sublingual and submandibular glands 47, 48. Amylase is a major of protein component of saliva, which is synthesized and secreted by acinar cells, a major cell population in the salivary gland. Enzymatic digestion of carbohydrates is the main function of saliva working through the amylase 49. In this study, we also determined the amylase activity of HSG cells treated with cordycepin. The higher level of amylase secretion in the HSG cells was found during 8-12 h co-culture in cordycepin while long-term culture period (1-5 day) stimulated lesser amount of amylase secretion. The pharmacokinetic research demonstrated that the half-life of cordycepin was short. Cordycepin is rapidly deaminated by adenosine deaminase and promptly metabolized to an inactive metabolite, 3'-deoxy-hypoxanthinosine 50, 51. It was found that cordycepin permeated the cell membrane of human endothelial-like immortalized cells (EA.hy926) cells within 30 min and was steady for a 3 h culture period 52. Although, cordycepin are rapidly reduced and metabolized in a short period of time, a cordycepin-induced unidentified compounds detected in blood and liver for over 2 h, which might have highly specific and widespread within the body organ or tissue 51, 53, 54. To overcome the problem of rapid elimination, a high concentration might be administered; otherwise, intracellular concentrations would be sub-therapeutic. Interestingly, we demonstrated that the amylase secretion was recovered after cordycepin treatment in HSG cells induced by H**_2_**O**_2_** as well as cordycepin significantly enhanced *AMY1A* and *AQP5* expression at both mRNA and protein levels. It is possible that recovering secretion of amylase under oxidative stress may be due to the ability of cordycepin on *AMY1A* gene and signaling pathways that are responsible for activating fluid and enzyme secretion 55. Furthermore, cordycepin probably promotes the effective function of *AQP5* gene which is the major pathway for regulating the salivary fluid secretion (water and protein) permeability in acinar cells 56, 57. Altogether, cordycepin could help cells restoring from oxidative stress by increasing salivary secretion via an increase in AMY and AQP5 protein levels and the resulting amylase secretion. However, despite the fact that we observed significant protein expression in the cordycepin-treated group compared to the untreated group, the enzyme activity was not dramatically different compared to the protein expression. This may be described by two explanations. First, the intracellular protein expression was analysed through immunofluorescence staining whereas the protein activity was evaluated from the supernated extracellular protein. Hence, the enzymatic function of secreted amylase may be affected by the post-modification or the formation of complex to function properly. Also, the functional amount of amylase in the supernatant may influence the amylase activity 58. Second, several previous studies have shown that the 2D *in vitro* model of salivary gland differentiation can enhance amylase expression and activity. However, the 3D model can better improve the differentiation. This latter model would help salivary gland cells to perform their function and activity better 59. Therefore, the 2D model that we selected in this study may limit the protein function.

## Conclusions

In conclusion, cordycepin could effectively protect against HSG cell death from H_2_O_2_-induced toxicity by reducing ROS generation via antioxidant and anti-apoptotic capacities. Furthermore, cordycepin exerts prevention by increasing both the expression of salivary marker genes and amylase secretion. Thus, these results suggest that cordycepin might be used as a new therapeutic agent and an experimental model for oral dryness.

## Figures and Tables

**Table 1:** PCR conditions and primers used in RT-PCR analysis

Genes	Direction	Primer sequences (5' → 3')	Annealing Temperature (°C)	Cycles	Product size (bp)
*AMY1A*	Forward	AATTGATCTGGGTGGTGAGC	52	35	474
	Reverse	CTTATTTGGCGCCATCGATG			
*AQP5*	Forward	CCTGTCCATTGGCCTGTCTGTCAC	56	40	249
	Reverse	GGCTCATACGTGCCTTTGATGATG			
*CAT*	Forward	TCCGGGATCTTTTTAACGCCATTG	55	30	362
	Reverse	TCGAGCACGGTAGGGACAGTTCAC			
*SOD1*	Forward	CTAGCGAGTTATGGCGAC	52	30	224
	Reverse	CATTGCCCAAGTCTCCCA C			
*GPX1*	Forward	CGCCAAGAACGAAGAGATTC	54	30	272
	Reverse	CAACATCGTTGAGACACA C			
*Caspase****-****3*	Forward	TTTGTTTGTGTGCTTCTGAGCC	54	40	279
	Reverse	ATTCTGTTGCCACCTTTCGG			
*BCL****-****2*	Forward	CGCATCAGGAAGGCTAGAGT	53	40	189
	Reverse	AGCTTCCAGACATTCGGAGA			
*GAPDH*	Forward	ACCTGACCTGCCGTCTAGAA	54	35	247
	Reverse	TCCACCACCCTGTTGCTGTA			
